# Severe Japanese Mamushi (*Gloydius blomhoffii*) bite

**DOI:** 10.1002/ccr3.1084

**Published:** 2017-07-30

**Authors:** Ezekiel Wong Toh Yoon, Yuichiro Otani, Syu Kabuto

**Affiliations:** ^1^ Department of Internal Medicine Hiroshima Kyoritsu Hospital Hiroshima Japan; ^2^ Department of Palliative Care Hiroshima Kyoritsu Hospital Hiroshima Japan

**Keywords:** Antivenom, Mamushi, snake bite

## Abstract

Venomous snake bites can be life threatening, occasionally requiring intensive care. For Mamushi bites, conservative treatment may be possible in mild cases but for severe cases or in cases where symptoms do not improve, a horse‐derived antivenom is indicated.

An otherwise healthy 84‐year‐old man was bitten by a Japanese Mamushi (*Gloydius blomhoffii*) on his left middle finger (Fig. 1A). At presentation, the swelling (and pain) was limited to his left hand, and laboratory data showed no abnormalities. By the following day, the swelling had spread to the entire left arm and axillary region (Fig. 1B), and he also complained of pain in his left chest. Electrocardiogram and chest X‐ray results were unremarkable.

**Figure 1:**
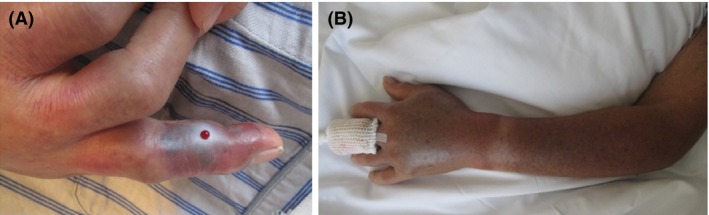
Photograph of the Mamushi bite site on the left middle finger (A) and the left arm (B) on the following day.

## What is the appropriate management for this case?

**Figure 2:**
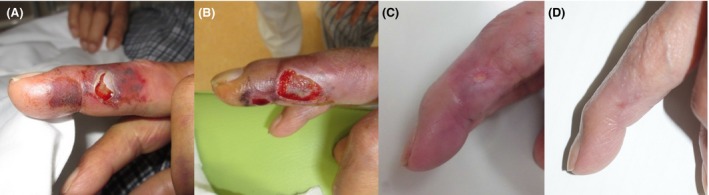
The bite site became necrotic (A) on day 6, requiring debridement (B). Wound healing observed two (C) and four (D) months later.

Corresponding to a severity of Grade III ‐ V (Severe) using a classification employed in Japan 1, he was given two doses of antivenom intravenously, after which his swelling and pain gradually receded. Although hepatic and renal function did not deteriorate, follow‐up laboratory examination revealed increased levels of creatine kinase (6295 U/L, Normal range: 62–287 U/L), further suggesting a severity of Grade V. Creatine kinase levels normalized as he improved, but the bite site became necrotic (Fig. 2A), requiring debridement (Fig. 2B). Marked healing was confirmed during a follow‐up two (Fig. 2C) and four months (Fig. 2D) later.

Mamushi is a pit viper responsible for 1.67 bites per 100,000 persons every 6 months and 10 deaths every year in Japan 1, 2. Severe cases of Mamushi bites usually require the administration of a horse‐derived antivenom 1.

## Authorship

EWTY: prepared the manuscript. YO and SK: had advisory roles in the management of the patient.

## Conflict of Interest

None declared.
